# Effect of the thin-film limit on the measurable optical properties of graphene

**DOI:** 10.1038/srep15684

**Published:** 2015-10-28

**Authors:** Jakub Holovský, Sylvain Nicolay, Stefaan De Wolf, Christophe Ballif

**Affiliations:** 1 Institute of Physics of the Academy of Sciences of the Czech Republic, Cukrovarnická 10, 162 00 Praha, Czech Republic; 2 CTU in Prague, Faculty of Electrical Engineering, Technická 2, 166 27 Prague, Czech Republic; 3 École Polytechnique Fédérale de Lausanne (EPFL), Institute of Microengineering (IMT), Photovoltaics and Thin-Film Electronics Laboratory, Maladière 71, CH-2000 Neuchâtel, Switzerland

## Abstract

The fundamental sheet conductance of graphene can be directly related to the product of its absorption coefficient, thickness and refractive index. The same can be done for graphene’s fundamental opacity if the so-called thin-film limit is considered. Here, we test mathematically and experimentally the validity of this limit on graphene, as well as on thin metal and semiconductor layers. Notably, within this limit, *all* measurable properties depend only on the *product* of the absorption coefficient, thickness, and refractive index. As a direct consequence, the absorptance of graphene depends on the refractive indices of the surrounding media. This explains the difficulty in determining separately the optical constants of graphene and their widely varying values found in literature so far. Finally, our results allow an accurate estimation of the potential optical losses or gains when graphene is used for various optoelectronic applications.

The discovery of free-standing graphene^1^ opened the fascinating field of two-dimensional material physics^2^,^3^,^4^,^5^. Since then, graphene’s transparency and exceptionally high carrier mobility have promised to revolutionize the field of thin-film optoelectronics^6^,^7^,^8^,^9^,^10^. Concerning the optical properties of graphene, the so-called thin-film limit (TFL) or thin-film approximation, obtained by taking the zero-thickness limit in classical formulae for the optical absorptance *A*, reflectance *R* and transmittance *T*, is frequently discussed^3^,^4^,^11^,^12^,^13^,^14^. Apart from graphene, the TFL has found applications in a variety of characterization methods, including differential reflectance spectrometry^15^ and infrared spectroscopy^16^,^17^, as well as in polarimetry of very thin layers^18^ and low absorptance spectroscopy^19^. In contrast to ultrathin atomic layers, their thicker counterparts requiring classical Fresnel formulae will be hereafter called macroscopically-thin layers. The remarkable consequences of the TFL appear if the layer is optically parameterized by its absorption coefficient *a*, thickness *d* and refractive index *n*^15^,^16^,^18^,^19^: (i) The measurable optical properties *A*, *R* and *T* do not depend on the parameters *a*, *d* or *n* individually, but only on their product *adn*. (ii) There is no dependency on the wavelength either, except through the dependencies of the parameters themselves. This explains why in the case of graphene—the thin film *par excellence*—considerable disagreement exists over the measured individual optical parameters^20^,^21^,^22^,^23^,^24^, and why there is some freedom in choice of assumed parameters, e.g. taking the diameter of valence orbitals or spacing of atomic planes in graphite as the thickness of graphene 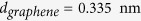
22,23,25,26,27, or equaling its refractive index to that of graphite 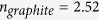
21,22,25. Actually, as argued by Chabal^17^, for an atomic monolayer, the thickness *d* and dielectric function *ε* lose their usual physical meaning and must rather be defined as tensors, relating to each other as 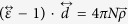. Here, the only parameters with physical meaning are *N* and 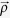, which are the dipole density and the vector of polarizability, respectively. Similarly, as shown already by Drude, the optical properties of an ultrathin film depend only on integral values of its dielectric function over the film thickness^28^. It was pointed out by Bruna and Borini^21^ that reflectance measurements of graphene can be—under some approximations—reproduced with an arbitrarily pre-defined value of constant refractive index.

The graphene’s *adn* product has been related to the fundamental sheet conductance 
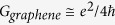
 (*e* being the electron charge and *ħ* the reduced Planck constant)^3^ by using the relation 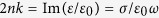, where 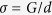 is the conductance, *ω* the angular frequency, 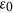
the vacuum permittivity and 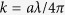 the extinction coefficient:


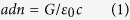


where *c* is the speed of light in vacuum. For graphene we obtain:


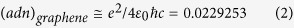


To analyze the effect of the TFL on graphene we take equations recently derived^19^, based on the conservation of energy, the continuity of the parallel components of an electric field across the layer, and the assumptions of a low-absorption medium (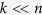) and a small thickness (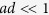, 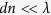). For perpendicular incidence, the following equations hold for absorptance *A*_TFL_, reflectance *R*_TFL_ and transmittance *T*_TFL_ of a layer between two media:


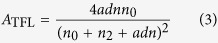



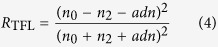



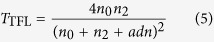


Here, 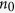, 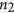 indicate respectively the refractive indices of the media over- and underlying the graphene layer. These equations can be converted to the ones typically found in literature, by normalization to the transmittance of the bare substrate^11^,^14^, by setting 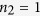, using sheet conductance *G* and vacuum impedance 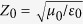
12,13 or by setting 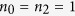 (i.e. a freestanding layer in air)^3^,^4^.

## Results

**Figure 1:**
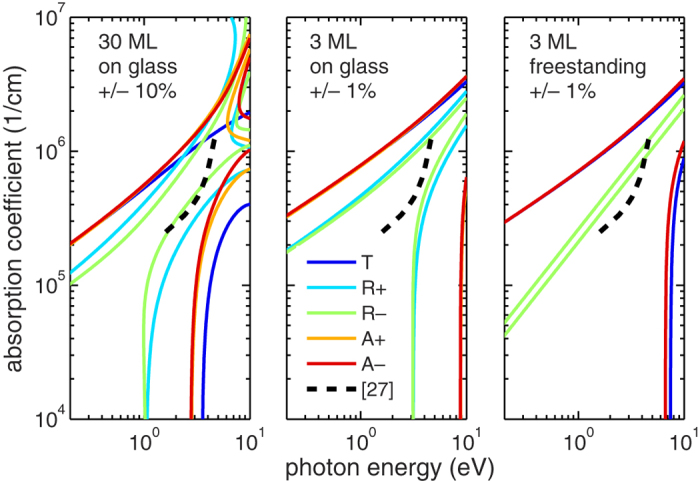
Lines represent contours between which the TFL differ from rigorous calculation less than 10% or 1% relatively. R+, A+, refer to incidence from layer side, conversely R–, A–, refer to glass side. Note the difference between freestanding layer and layer on glass. Dashed line between 1.6 eV and 5 eV indicates the absorption coefficient taken from ref. 27.

We first numerically investigate the range of validity of the TFL by comparison to rigorous Fresnel formulae. Figure 1 shows contour plots between which the error of TFL is less than 10% or 1%. The abscissae display the spectral dependence in photon energy; its logarithmic scale deliberately extended to 10 eV to show more complete picture. The ordinates show the absorption coefficient of a hypothetical material with thickness corresponding to 3 or 30 monolayers (ML) of graphene and with constant refractive index 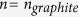.

In a first case we analyze 30 monolayers (ML) on glass and investigate regions of validity within 10% accuracy. The validity regions are in general limited by high energy and high absorption coefficient thresholds stemming from the above mentioned assumptions: 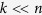, 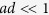, 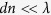. Additionally, there is a tendency to limit the region to the area close to a line satisfying approximately the relation 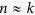, approaching the case of a purely imaginary permittivity. Considering the transmittance (violet) and absorptance (red, yellow) only, the TFL is—for reference data of 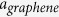 taken from ref. 27 (dashed line)—valid in whole its range from 1.6 eV to 5 eV. When additionally the reflectance (green, blue) is considered, the validity region shrinks, yet only the range from 1.8 eV to 3.6 eV falls outside this region and only for glass-side incidence. 

In a second case we consider a 10× thinner sample (3 ML), 10× better accuracy (1%) and we obtain slightly broader regions of TFL validity than in the previous case. In this case, the reference data of 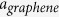 fall completely into the region of validity. This implies that when measuring less than 3 graphene monolayers on glass under perpendicular incidence, in the range up to 5 eV with 1% relative accuracy, one *cannot* distinguish between absorption coefficient, refractive index and thickness. This is valid in the near-infrared to visible range for any material with absorption coefficient below 10^5^cm^−1^.

In a third case, we remove the glass substrate, assuming thus a freestanding layer. The region of TFL validity for transmittance and absorptance changes slightly, but for reflectance, conversely, the validity of TFL shrinks to a negligible region around the 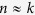 line. The reference 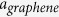 satisfies the validity only in the range from 4.2 eV to 4.6 eV. This means that measuring the reflectance of freestanding layer is a way to avoid the TFL, enabling improved distinction between *a*, *d* and *n*. For oblique incidence, additional simulations (not shown here) prove a similar difficulty to distinguish between *a*, *d* and *n*, for thin layers on a substrate. However, angles far from normal incidence, as in ellipsometry, always increase significantly the ability to distinguish between these parameters.

Experimentally, the validity of the TFL can be verified independently from the actual value of the *adn* product, thanks to one of the consequences of the TFL: The values *A*_TFL_*, R*_TFL_ and *T*_TFL_ are mutually dependent in such a way that by measuring only one of them we can calculate the remaining two. By combining equations (4) and (5), one obtains for a layer on an interface:


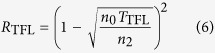


Knowing *R*_TFL_ and *T*_TFL_, *A*_TFL_ is calculated as 1−*R*_TFL_−*T*_TFL_. Noteworthy, this yields a ‘universal’ relation that applies to materials beyond graphene.

**Figure 2:**
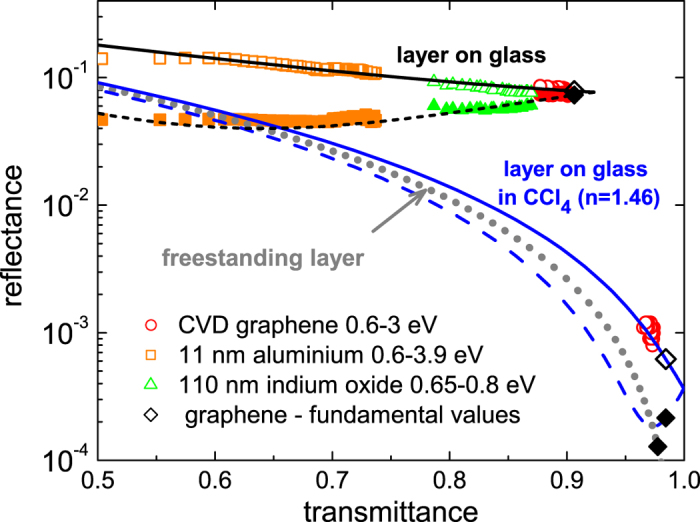
Lines: universal relationship between *T*_TFL_ and *R*_TFL_ in the range 0.7–3 eV of a freestanding layer (dotted line) and of a layer on glass in air or in CCl_4_ (full and dashed lines are for layer-side and glass-side, respectively). Symbols: theoretical and experimental values for different materials (full and empty symbols are for glass-side and layer-side, respectively).

For any value of the *adn* product, we simulate in Fig. 2 the relationship between *R*_TFL_ and *T*_TFL_: according to (6) for a freestanding layer, and according to (4) and (5), while accounting multiple reflections for the case of the layer on glass. The latter is also simulated for the case of immersion in carbon tetrachloride (CCl_4_). The advantage of the CCl_4_ is that at room temperature its refractive index is similar to the one of glass. As such, the situation of freestanding layers can be approached. Black symbols show the theoretical *T* vs. *R* relations, when graphene’s fundamental conductivity (2) is taken. To compare with experiments, the pairs of transmittance and reflectance values represent points in the graph, plotted by symbols. We see that the symbols for graphene fall well on the theoretical curves. In addition, the TFL was equally well fulfilled for an 11-nm-thick layer of evaporated aluminum over a broad spectral range, and also for a 110-nm-thick indium oxide layer, but only in the infrared region (<0.8 eV).

**Figure 3:**
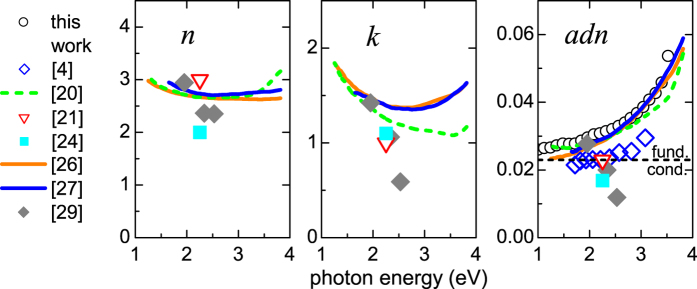
Different values of *n* and *k* spectra of graphene found in the literature (references in square brackets) and the respective calculated *adn* products. The dashed line on the right shows the fundamental value given by equation (2) from fundamental conuctivity. Circles represent the data experimentally obtained on the sample of CVD graphene.

The absorptance of graphene monolayer, measured with high accuracy by photothermal deflection spectroscopy was then used to evaluate the *adn* product from equation (3). This *adn* product is shown in Fig. 3 together with *n* and *k* spectra of single-layer graphene, taken from literature^4^,^20^,^21^,^24^,^26^,^27^,^29^. This graph demonstrates that there is a larger discrepancy among the published *n* and *k* values of graphene samples, compared to their respective *adn* products. This is consistent with the fact that graphene on a substrate (measured in transmission and reflection) and freestanding graphene (when measured in transmission only) always fulfills the TFL over a broad wavelength range (see Fig. 1), and that the separation of the optical constants is difficult. Measurement of reflectance of a freestanding or embedded layer is therefore recommended.

Finally, we evaluate, based on the TFL, the losses or gains of using graphene as transparent functional layer. It follows from equation (3) that the absorptance of any ultrathin layer can be reduced by embedding it into a high-refractive-index medium or by depositing it on high-refractive-index substrate. However, in the latter case, as expected, the transmittance will also be reduced due to the increased reflectance at such a substrate. So, in order to assess how the absorptance is reduced due to the TFL, it is convenient to normalize *A* by *T*. The ratio *A*/*T* then characterizes the fraction of light that is absorbed during transmission, establishing a useful measure for the window-material performance. It follows from (3) and (5) that for an ultrathin layer on a substrate or a freestanding layer:


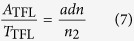


Moreover, the *A*/*T* ratio is also a good parameter for evaluating macroscopically-thin layers, because for a layer on a substrate the *A*/*T* ratio is virtually free from interference effects and free from direct wavelength dependencies^30^, being therefore perfect for comparison to equation (7).

**Figure 4:**
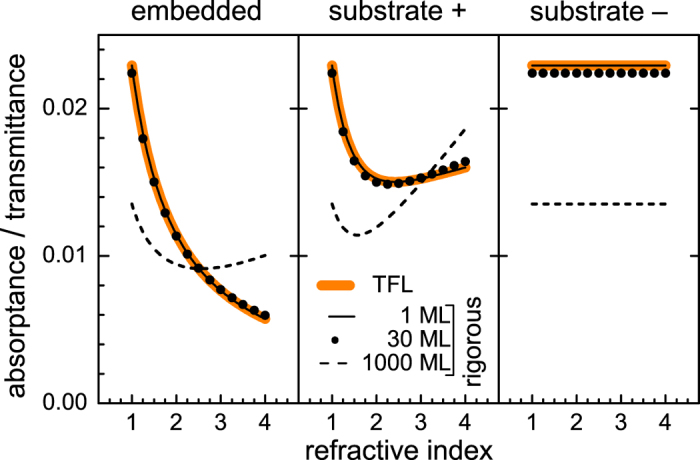
The effect of increase of refractive index of surrounding medium or substrate on the *A*/*T* ratio at photon energy 2.25 eV. “+” refers to incidence from layer-side, conversely, “−” refers to substrate-side. The cases with different thicknesses have the same *adn* product.

In Fig. 4 we simulated for a single photon energy (2.25 eV) the *A*/*T* ratio of a layer on a finite substrate by TFL and rigorously. In both cases the effect of multiple reflections in the substrate is accounted for by the Fresnel equations. We tested a set of thicknesses and absorption coefficients while keeping the value of *adn* product fixed to 0.0229. Three cases were considered: the embedded layer, the layer-on-substrate for layer-side incidence and the layer-on-substrate for substrate-side incidence. We see that for the embedded layer, as well as for the layer-on-substrate, the increase of refractive index of the surrounding medium or the substrate can indeed significantly reduce the ratio *A*/*T*. As long as the TFL describes well this phenomenon (well up to a film thickness of 10 nm) it is advantageous to embed graphene in, or place it on top of, a high-refractive-index medium. For macroscopically-thin layers (e.g. in our case 335 nm) this trend is weakened, and importantly, for lower values of refractive index of the surrounding the *A/T* ratio of macroscopically-thin layer is lower than that of ultrathin layer. This implies, that thinning down a layer while keeping the *adn* product constant is not favorable, unless a high-refractive-index surrounding medium, e.g. silicon, is used. Interestingly, for the substrate-side incidence the refractive index has no effect on *A/T* ratio. These effects are crucial when comparing optoelectronic applications of graphene with usual macroscopically-thin window layers.

## Discussion

Within a given spectral region and depending on the substrate and incidence angle, thin layers may satisfy the thin-film limit when their measurable optical properties are given only by the product of *a, d*, and *n*. Graphene satisfies this limit over a broad spectral range and it makes the separate determination of its optical constants difficult, especially when graphene is on a substrate. The layer thickness, as a condition of the limit, should rather be compared to the vacuum wavelength; in the infrared and upon perpendicular incidence, the limit can be satisfied even by a 100-nm-thick layer on glass (e.g. of indium oxide below 0.8 eV). Within the thin-film limit, the plot of reflectance versus transmittance is, for a given surrounding medium, a universal curve, which was also used here for experimental verification. Another interesting quantity is the absorptance normalized to transmittance, which is perfectly suitable for comparing absorption losses in graphene and other window layers. It shows that if the thin-film limit is satisfied, the performance is strongly enhanced by the high refractive index of the underlying medium.

## Methods

The transmittance-reflectance spectroscopy was done either in air by Perkin-Elmer Lambda 900 or in a carbon tetrachloride (CCl_4_) in a custom-made setup. Our custom-made setup also allows photothermal deflection spectroscopy (PDS)^31^ measurements of absorptance with sensitivity down to 10^−4^ through heating of immersion liquid, e.g. CCl_4_. The refractive index of CCl_4_ is around 1.46 in our spectral range (0.6–3 eV)^32^. For our tests, we used a single layer of chemical-vapor-deposited (CVD) graphene on borosilicate glass obtained from https://graphene-supermarket.com/Transparent-Conductive-Coatings/. We also used a layer of aluminum, thermally evaporated at pressure 5×10^–5^ mbar and a layer of indium oxide, sputtered in DC regime at 6 mbar^33^. In both cases the Schott AF45 low-alkaline borosilicate glass served as a substrate.

## Additional Information

**How to cite this article**: Holovský, J. *et al.* Effect of the thin-film limit on the measurable optical properties of graphene. *Sci. Rep.* **5**, 15684; doi: 10.1038/srep15684 (2015).
